# Lived experiences of sexual wellbeing and psychosexual needs among adolescents and young adults with sarcoma: a qualitative study

**DOI:** 10.3389/fpsyg.2026.1866967

**Published:** 2026-08-21

**Authors:** Gabriella Maggi, Anita Caruso, Denise Minghelli, Lara Guariglia, Maria Perrone, Roberto Biagini, Giuseppe Francesco Papalia, Virginia Maria Formica, Virginia Ferraresi, Sabrina Vari, Concetta Elisa Onesti, Nicolò Panattoni, Fabrizio Petrone, Aurora De Leo

**Affiliations:** 1 Psychology Unit, IRCCS Regina Elena National Cancer Institute, Rome, Italy; 2 Orthopaedic Oncology Unit, IRCCS Regina Elena National Cancer Institute, Rome, Italy; 3 UOSD Experimental Clinic for Sarcomas, Rare Cancers and Metastatic Melanomas, Rome, Italy; 4 Nursing Research Unit IFO, IRCCS Regina Elena National Cancer Institute, Rome, Italy

**Keywords:** adolescent, educational needs, qualitative research, sarcoma, sexual wellbeing, young adult

## Abstract

**Introduction:**

Sexual wellbeing is an important yet often overlooked aspect of quality of life for many Adolescent and Young Adult patients with sarcoma. Given the significant impact of this issue and the dearth of previous research, this study aims to explore the sexual wellbeing and psychosexual needs of this group.

**Materials and methods:**

A descriptive, phenomenological, qualitative study was conducted. Open-ended, face-to-face interviews were used to achieve data saturation, following Giorgi’s approach. The Lincoln and Guba criteria and the COREQ guidelines were used to enhance methodological rigor and reporting quality. For descriptive purposes, information was collected on sociodemographic, clinical, quality of life and patient distress issues. Given the complexity of obtaining informed consent from parents or legal guardians of minors, the study included participants between the ages of 18 and 39 at the time of data collection.

**Results:**

Twenty interviews with patients aged 18–39 years were collected. One overall domain, five themes, and 21 related sub-themes emerged from the thematic analysis. They were mainly related to the impact of the cancer diagnosis on patients’ priorities, scars and their symbolism and relationships with healthcare professionals and partners to promote sexual wellbeing. Patients reported relatively low levels of symptoms and high levels of functioning. The main symptom was insomnia, whereas fatigue and pain were less severe. Slight impairments were observed in the subject’s emotional functioning. 70% of patients had clinically significant Distress (≥4).

**Discussion:**

Cancer significantly impacts the emotional relationships and sexual health of patients. A lack of information and taboos surrounding sexuality in oncology are significant barriers to patients’ psychological and physical wellbeing. Psychologists and nurses, in particular, should implement strategies to encourage open discussion about sexuality within this patient group.

## 1 Introduction

Sarcomas are a heterogeneous group of neoplasms account for less than 1% of all solid neoplasms, occur in all anatomical locations and affect all age groups (Bray et al., 2024). In particular, bone sarcomas such as osteosarcoma and Ewing’s sarcoma occur most frequently in Childhood, Adolescence and Young Adulthood (AYA), accounting for around 11% of all cancers in this population (Drabbe et al., 2021). Multimodal treatment in patients with sarcoma consists of a combination of surgical excision of the primary neoplasm, chemotherapy before surgery (neoadjuvant), chemotherapy after surgery (adjuvant), and/or radiotherapy (Gronchi et al., 2021). Specifically, the side effects of cancer treatments in young adults with sarcoma depend on the type of sarcoma, anatomical location, chemotherapy protocol, and radiation dose and location. The most common side effects of chemotherapy include fatigue, sexual and fertility problems, gastrointestinal disturbances, alopecia, and cardiotoxicity. Similarly, the most common side effects associated with radiation therapy include fatigue, skin reactions, edema, and infertility due to pelvic radiation. Furthermore, the frequently destructive nature of surgery can cause significant psychological and physical disabilities in survivors (DeVine et al., 2025; Ferrari et al., 2021).

In this regard, AYA patients aged 18–39 years face these potentially life-threatening cancers and treatments at a challenging and critical time in their emotional, cognitive and social development (Ferrari et al., 2021). In particular, in AYA patients with bone sarcomas, treatment-related changes, such as hair loss, weight changes, extensive scarring, amputations and infertility, can negatively impact body image, self-esteem, and psychological adaptation (Kosir et al., 2020). Consequently, these changes can potentially affect their quality of life (QoL) and survival (Soanes and White, 2018). Furthermore, sexual health is also a sensitive issue for this population, which can be affected by the toxic effects of surgical, chemotherapy, and radiotherapy treatments, even compromising fertility (Gunderson et al., 2025). This topic is of considerable importance for its psychosocial impact on personal autonomy, intimate relationships and body image during a crucial phase of life for identity construction, causing specific unmet needs (Cherven et al., 2024; Neylon et al., 2023).

Recent studies have focused on the sexual needs of AYA patients, highlighting the lack of information from healthcare professionals, focusing their attention mainly in the context of reproductive cancers and infertility (Mütsch et al., 2019). Although the clinical guidelines (DeVine et al., 2025) recommend discussing sexual wellbeing with these patients, there is currently a lack of psychosexual interventions, standardized communication strategies, and screening tools for addressing these issues within the care pathways of AYAs. In particular, the unmet needs of AYA cancer patients relating to contraception, infection prevention and sexual function are particularly challenging (Cherven et al., 2024). On the contrary, adequate information would allow them to feel more involved in therapeutic choices and more willing to face the side effects of treatments and their impact on sexual function. Although sexual dysfunction affects 50–70% of cancer survivors, it is still underestimated and overlooked by healthcare professionals (Alqaisi et al., 2025). In contrast, the health outcomes for this population can be significantly improved by trained oncology nurses and psychologists meeting their psychosexual needs and supporting them in coping with their condition.

Due to the significant impact of treatment on the sexual wellbeing and psychosexual needs of AYA patients with sarcomas (Ferrari et al., 2018), and the lack of specific studies in this population, this study aims to explore their experiences and needs in this area using a qualitative approach (Sandelowski, 2000). In line with Lehmann’s previous research in this area (Lehmann et al., 2022), the study focused on the AYA age group 18–39 years.

## 2 Materials and methods

A qualitative, monocentric, descriptive study (Sandelowski, 2000) was conducted using a phenomenological approach from Husserl’s perspective (Husserl, 1967) and Giorgi’s approach (Giorgi, 2009). For descriptive and contextual purposes, the main socio-demographic and clinical characteristics of the participants were collected, alongside their perceptions of health-related QoL and Distress. Quantitative data on patients’ perceived QoL and their level of distress was collected using the Quality of Life Questionnaire of the European Organization for Research and Treatment of Cancer, EORTC QLQ-C30 (Aaronson et al., 1993) and the Distress Thermometer (Boyes et al., 2013). The EORTC QLQ-SH22 module (Oberguggenberger et al., 2024), which focuses on the impact of curative cancer treatment on sexual health, was not used in this study. This choice was dictated by the lack of specific validation of the QLQ-SH22 questionnaire on the study population (AYA patients with cancer aged 18–39 years).

As the qualitative and quantitative data were not formally integrated, the study did not adopt a mixed-methods approach (Creswell and Plano Clark, 2023).

In-depth, open-ended face-to-face interviews were used to explore patients’ lived experiences (Husserl, 1967). The Lincoln and Guba criteria (Lincoln and Guba, 1985) and the COnsolidated Criteria for Reporting Qualitative Research (COREQ) (Tong et al., 2007), were used to enhance, respectively, the rigor and quality of the study, and reporting.

### 2.1 Ethical considerations

The local Ethics Committee approved the phenomenological study on 20 December, 2023 (protocol number 71/IRE/23). During follow-up visits, the team members introduced the study to all eligible patients. The researchers who conducted the interviews explained the aims, objectives and procedures of the study to all interested patients. Participation was free and voluntary, preceded by the signing of a specific written informed consent form. Prior to the interviews, the researchers established a relaxed and trusting relationship with the respondents. All the interview questions and items from the EORTC QLQ-C30 (Aaronson et al., 1993) and the Distress Thermometer (Boyes et al., 2013), had previously been explained and read by the respondents. A debriefing session was held at the end of the interviews to assess the impact on patients and healthcare professionals. The study procedures, particularly the interviews, were carried out in a comfortable environment. All interviews were audio-recorded with the consent of the participants. The data were analyzed and summarized in an anonymous, aggregated form.

### 2.2 Study setting and recruitment

Convenience sampling with maximum variation and extreme case sampling were used to identify the experiences of AYA patients with sarcoma on the phenomenon (Tuckett, 2004; Sandelowski, 1995). Specifically, the study was presented to each AYA patient during follow-up visits to the dedicated sarcoma outpatient clinic of an Italian National Cancer Centre by the team members of the study, between February and July 2024. Before the interview, interested patients were informed about the study’s aims and their participation was confirmed by signing an informed consent form.

Following the sampling methodology for qualitative research (Tuckett, 2004, Sandelowski, 1995), the following criteria were used for recruitment:

*Inclusion criteria*: patients undergoing follow-up after surgical, chemotherapy and radiotherapy treatments; patients willing to comply with study procedures and speak Italian. In line with Lehmann’s previous research in this area (Lehmann et al., 2022), and given the complexity of obtaining informed consent from parents or legal guardians of minors, the study included participants between the ages of 18 and 39 at the time of data collection the present study did not include adolescents < 18 years old. The differences in experiences and perceptions of sexuality within this population, as well as the requirement for parental consent for treatment, were deemed to warrant a separate study. Nevertheless, subjects who were < 18 years old at the time of diagnosis and during the treatment periods that were reconstructed during the interviews, were included in this study.

*Exclusion criteria*: patients with different neoplasms and age; patients undergoing chemotherapy or radiotherapy at the time of enrolment in the study; patients unable to understand or speak Italian, or with cognitive impairment.

### 2.3 Data collection and used tools

Prior to the interviews, a questionnaire was used to collect key sociodemographic and clinical data on the patients involved in the study. This included information on their age, gender, marital status, level of education, employment status, the number and type of family members, the type and date of their cancer diagnosis, and any previous or current surgical, chemotherapy or radiotherapy treatments they had received.

The EORTC QLQ-C30 questionnaire (Aaronson et al., 1993) and the Distress Thermometer (Boyes et al., 2013) were used to assess patients’ QoL and wellbeing, with descriptive and contextual purposes.

The EORTC QLQ-C30 questionnaire (Aaronson et al., 1993) is a validated, self-administered tool used to collect information on health-related QoL in cancer care. It consists of 30 items that assess five functional domains (physical, role, emotional, cognitive and social functioning), the main symptoms and syndromes experienced by cancer patients (fatigue, pain and nausea/vomiting, dyspnea, loss of appetite, sleep disturbance, constipation, diarrhea, financial impact), perceived overall health and QoL. The symptom scale ranges from 1 (no symptoms) to 4 (high burden), while the overall health and overall QoL scales range from 1 (very poor) to 7 (excellent). The EORTC QLQ-C30 scores were converted into a linear scale ranging from 0 to 100 (Aaronson et al., 1993). Better functioning and higher QoL are indicated by higher scores. Greater symptom burden is indicated by higher scores on symptom scales.

The Distress Thermometer (Boyes et al., 2013) is a validated, self-administered tool used to assess patient distress in various areas, such as physical health, spirituality, emotions, relationships, and daily life issues. This screening tool is frequently used in cancer care. It assesses patients’ perceived distress over the past 7 days on a scale from 0 (no distress) to 10 (extreme distress). According to the authors (Boyes et al., 2013), a score ≥ 4 suggests significant distress in the patient and usually suggests a psychology consultation.

The seven open-ended questions that guided the interviews were summarized from a preliminary literature review conducted by the authors on the topic (Lehmann et al., 2022; Veneroni et al., 2020). The questions mainly focused on the lived experience of sexuality of AYA patients with sarcoma throughout their care pathway, their psychosexual needs and the role of healthcare professionals and partners (Table 1).

**Table 1:** The interview.

Key topics	Questions
The experience of sexuality in the care pathway	During your treatment, have you had the opportunity to discuss sexuality with your treatment team?
When and with whom to talk about sexuality	Could you describe how you experienced sexuality during your treatment?
	With which health professional (s) have you discussed your sexuality, or would you like to?
	In your experience, at different stages of your treatment pathway, is there a better time than others (right) to talk about sexuality?
	Do you think it would be useful to talk to health professionals about the effects of the disease and medical treatments on sexuality?
Partner involvement: a facilitator or an obstacle to communication about sexuality?	Do you think it would be helpful to involve your partner when discussing sexuality?
	Do you think your partner’s presence at the follow-up visit helps or hinders communication about sexuality?

Following Giorgi’s (Giorgi, 2009) and Husserl’s perspective (Husserl, 1967), open-ended individual interviews lasting up to 20 min were conducted by psychologists or nurses (G. M., D. M., N. P., F. P., and A. D. L.: three females and two males) to explore patients’ experiences. With the patient’s consent, the interviews were conducted and audio recorded in a confidential setting at the National Cancer Centre. Interviews continued until data saturation was reached, as indicated by a lack of new information and redundant data on the topic (Tuckett, 2004; Sandelowski, 1995). In order to improve the quality of the thematic analysis (Braun and Clarke, 2024), patients were encouraged to provide broad, detailed and rich descriptions of their lived experience. According with the phenomenological approach, data collection and analysis were carried out simultaneously until data saturation (Tuckett, 2004, Sandelowski, 1995).

### 2.4 Data analysis

In order to enable thematic analysis (Braun and Clarke, 2024), the recorded interviews were transcribed verbatim, meaning that the patients’ words were transcribed exactly as they were spoken. In order to understand the description’s overall meaning, the researchers read the transcripts several times, allowing the data to speak for itself without categorizing the information (Giorgi, 2009). Giorgi’s approach (Giorgi, 2009) and thematic analysis (Braun and Clarke, 2024) were used to summarize the transcripts in tables. Similar “units of meaning” with a proper meaning were then aggregated into subcategories, which were grouped into broader categories. This process was undertaken to identify the deeper meaning of patients’ experiences as expressed through themes and sub-themes (Giorgi, 2009). Data collection and analysis were conducted simultaneously by experienced researchers in qualitative research (G. M., D. M., G. F. P., V. M. F., C. E. O., N. P., F. P. and A. D. L.), until data saturation (Tuckett, 2004; Sandelowski, 1995).

Descriptive statistics were used to summarize the socio-demographic and clinical characteristics of the patients, as measured using the data collection form and the EORTC QLQ-C30 questionnaire (Aaronson et al., 1993), as well as the Distress Thermometer (Boyes et al., 2013). Absolute and relative frequencies were used to report categorical variables, while means and standard deviations (SD) were used for continuous variables.

SPSS, Statistical Package for Social Science 29.0 software (Chicago, IL), was used for data analysis.

### 2.5 Rigor and reflexivity

The researchers who conducted the interviews (G. M., D. M., A. D. L., F. P. and N. P.: three females and two males) were trained in qualitative research methods to improve their reflexivity and were experienced psychologists and nurses specializing in cancer care. In this regard, according to phenomenology (Husserl, 1967) and Giorgi’s approach (Giorgi, 2009) during the data collection and interpretation, the researchers held meetings to assess whether their own impressions or preconceptions were influencing their work (bracketing and reflexivity). The authors involved in the data collection and analysis (G. M., D. M. G. F. P., V. M. F., C. E. O., N. P., F. P., and A. D. L.: five females and three males), coded the transcripts independently. Any discrepancies were resolved through discussion until consensus was reached. The multidisciplinary team, comprising psychologists, nurses, surgeons and oncologists with expertise in this area, was dedicated to gaining a comprehensive understanding of the lived experiences of sexual wellbeing and psychosexual needs among AYA patients with sarcoma.

To improve data quality and reflexivity, extreme case and maximum variance sampling, researcher diaries, data and researcher triangulation were used (Tuckett, 2004; Sandelowski, 1995). In addition to the Lincoln and Guba criteria (Lincoln and Guba, 1985), (credibility, dependability, confirmability and transferability), data saturation, peer review, debriefing and supervision by experienced qualitative researchers (A. D. L. and N. P.) were used to enhance the quality and the rigor of the study.

## 3 Results

Twenty patients with sarcoma were included in the phenomenological study using convenience sampling until data saturation (Tuckett, 2004, Sandelowski, 1995).

### 3.1 Characteristics of participants

Tables 2, 3 show the main socio-demographic and clinical characteristics of the sample. No patients withdrew or repeated interviews during the study. Two patients (one male and one female) expressed interest in the study, but refused to enroll due to a lack of time.

**Table 2:** Sociodemographic characteristics of the sample.

Characteristics	Median (range)
Age (mean)	29.5 (21–38)
Gender	
Male	13 (65%)
Female	7 (35%)
Education	
Primary school	0 (0%)
Middle school	1 (5%)
High school	10 (50%)
Degree	9 (45%)
Marital status	
Single	13 (65%)
Married/Living with a partner	7 (35%)
Divorced/Separated	0 (0%)
Widow	0 (0%)
Employment	
Yes	12 (60%)
No	8 (40%)
Lives alone	
Yes	1 (5%)
No	19 (95%)
Cohabitation with parents	
Yes	9 (45%)
No	11 (55%)
Cohabitation with partner	
Yes	11 (55%)
No	9 (45%)
Cohabitation with son/daughter	
Yes	3 (15%)
No	17 (85%)
Son/daughter (mean)	3 (15%)

**Table 3:** Clinical characteristics of the sample.

Characteristics	Median (range)
Cancer	
Synovial Sarcoma	4 (20%)
Osteosarcoma	4 (20%)
Sarcoma Ewing	4 (20%)
Giant Cell Tumor	1 (5%)
Myxoid Liposarcoma	2 (10%)
Clear Cell Sarcoma	1 (5%)
Undifferentiated Pleomorphic Sarcoma	1 (5%)
Sarcoma	1 (5%)
Spindle-Cell Sarcoma	1 (5%)
Epithelioid Sarcoma	1 (5%)
Surgical treatment	
Yes	20 (100%)
No	0 (0%)
Type of surgery	
Limb sparing	19 (95%)
Amputation	1 (5%)
Localization	
Upper limb	4 (20%)
Lower limb	15 (75%)
Other	1 (5%)
Chemotherapy	
Yes	17 (85%)
No	3 (15%)
Radiotherapy	
Yes	8 (40%)
No	12 (60%)
Current treatment	
Follow-Up	20 (100%)

All enrolled patients completed the EORTC QLQ-C30 questionnaire (Aaronson et al., 1993) and the Thermometer Distress (Boyes et al., 2013) (Table 4).

**Table 4:** Symptoms’ and functional scales.

Symptom	Median (range)
Dyspnea	4.95 (0–33)
Pain	11.65 (0–67)
Fatigue	17.6 (0–44)
Insomnia	23.2 (0–100)
Appetite loss	0 (0–0)
Nausea	2.55 (0–17)
Constipation	4.95 (0–33)
Diarrhea	4.95 (0–33)
Functional scale	Median (range)
Physical	84.35 (60–100)
Role	80.8 (17–100)
Emotional	76.3 (42–100)
Cognitive	90.75 (67–100)
Social	84.1 (33–100)

The functional scales analysis suggested that patients experienced improved physical, social and cognitive wellbeing, but poorer emotional and role functioning (Aaronson et al., 1993). Analysis of symptom scales showed insomnia, fatigue, and pain, impacted QoL the most. Distress scores (Boyes et al., 2013) showed 70% of subjects had clinically significant levels (≥4).

### 3.2 Thematic analysis: themes and sub-themes

**Figure 1:**
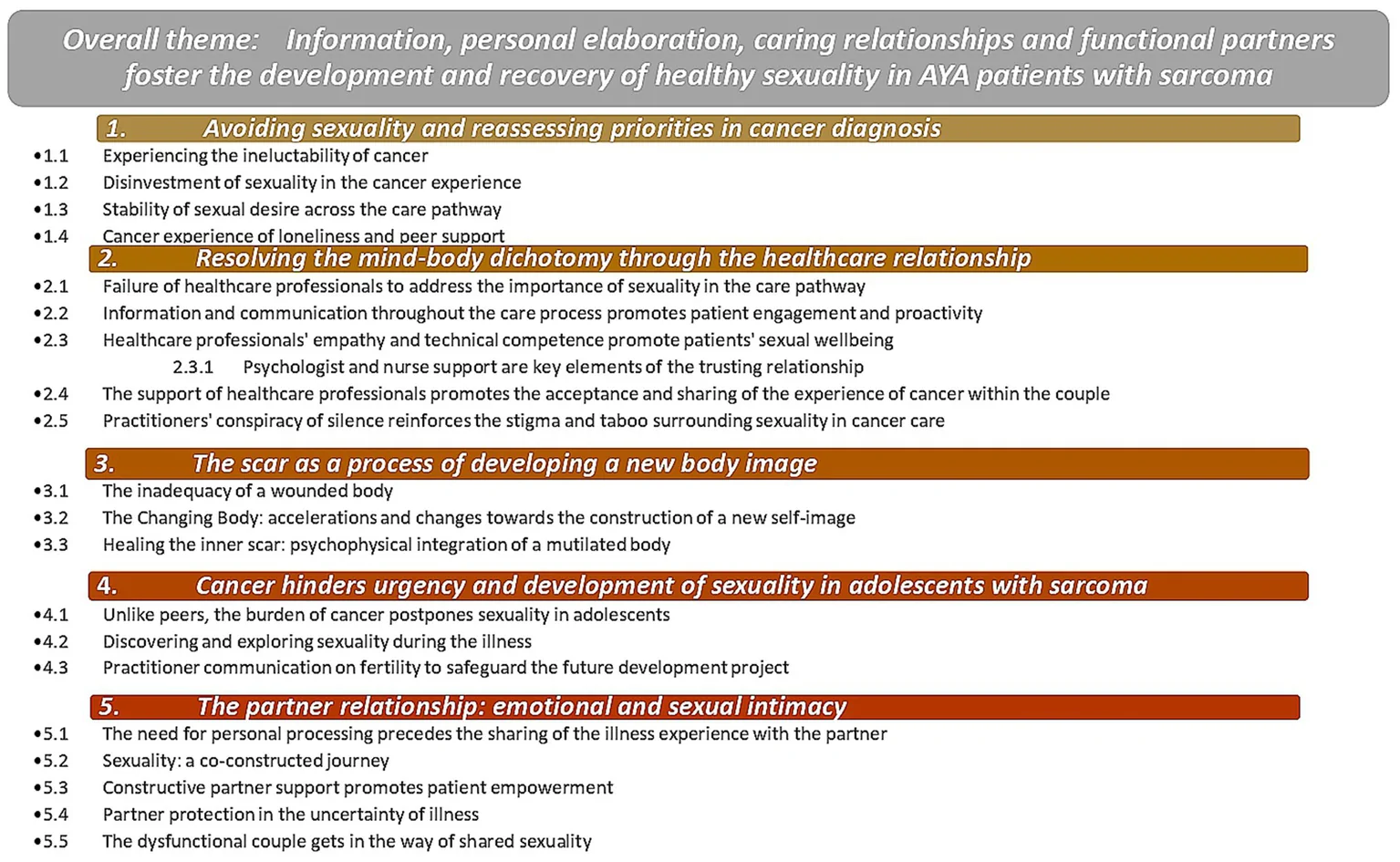
Themes and sub-themes.

In accordance with the qualitative approach and thematic analysis, data collection and analysis were concluded when thematic and meaning redundancies emerged from the interviews (data saturation) (Tuckett, 2004, Sandelowski, 1995). This point was considered the moment when the interviews did not reveal new information useful for developing the thematic analysis. Qualitative analysis (Braun and Clarke, 2024; Giorgi, 2009) highlighted one overall domain, five themes and 21 one associated sub-themes (Figure 1; Table 5).

**Table 5:** Prevalence of themes and sub-themes in the interviews.

Overall theme	Theme	Sub-theme	Patient
Information, personal elaboration, caring relationships and functional partners foster the development and recovery of healthy sexuality in AYA patients with sarcoma	1. Avoiding sexuality and reassessing priorities in cancer diagnosis	*1.1 Experiencing the ineluctability of cancer*	P1, P4, P8, P9, P10, P11, P12, P13, P14, P15, P16, P18, P20.
		*1.2 Disinvestment of sexuality in the cancer experience*	P1, P2, P3, P4, P6, P11, P12, P14, P16, P17, P18, P19.
		*1.3 Stability of sexual desire across the care pathway*	P1, P4, P5, P8, P9, P10, P14, P19.
		*1.4 Cancer experience of loneliness and peer support*	P1, P3, P6, P8, P11, P18, P19.
	2. Resolving the mind–body dichotomy through the healthcare relationship	*2.1 Failure of healthcare professionals to address the importance of sexuality in the care pathway*	P1, P5, P6, P8, P9, P11, P12, P13, P15, P20.
		*2.2 Information and communication throughout the care process promotes patient engagement and proactivity*	P1, P2, P3, P4, P5, P7, P8, P9, P11, P14, P15, P16, P17, P18, P19, P20.
		*2.3 Healthcare professionals’ empathy and technical competence promote patients’ sexual wellbeing*	P5, P6, P7, P8, P10, P12, P14, P15, P16, P20.
		*2.3.1 Psychologist and nurse support are key elements of the trusting relationship*	P2, P4, P5, P6, P7, P8, P9, P11, P12, P14, P15, P16, P17, P18, P20.
		*2.4 The support of healthcare professionals promotes the acceptance and sharing of the experience of cancer within the couple*	P2, P3, P7, P9, P13, P17, P20.
		*2.5 Practitioners’ conspiracy of silence reinforces the stigma and taboo surrounding sexuality in cancer care*	P3, P6, P7, P9, P13, P18.
	3. The scar as a process of developing a new body image	*3.1 The inadequacy of a wounded body*	P3, P9, P10, P15, P18.
		*3.2 The Changing Body: accelerations and changes toward the construction of a new self-image*	P3, P5, P7, P9, P8, P15.
		*3.3 Healing the inner scar: psychophysical integration of a mutilated body*	P2, P11.
	4. Cancer hinders urgency and development of sexuality in adolescents with sarcoma	*4.1 Unlike peers, the burden of cancer postpones sexuality in adolescents*	P3, P4, P6, P7, P17.
		*4.2 Discovering and exploring sexuality during the illness*	P1, P2, P9, P16.
		*4.3 Practitioner communication on fertility to safeguard the future development project*	P6, P9, P17, P18, P20.
	5. The partner relationship: emotional and sexual intimacy	*5.1 The need for personal processing precedes the sharing of the illness experience with the partner*	P1, P5, P7, P9, P10, P11, P12, P14, P15, P18.
		*5.2 Sexuality: a co-constructed journey*	P2, P3, P4, P5, P6, P7, P8, P9, P11, P12, P13, P16, P20.
		*5.3 Constructive partner support promotes patient empowerment*	P2, P3, P7, P10, P13, P15, P17.
		*5.4 Partner protection in the uncertainty of illness*	P3, P5, P12, P19, P20.
		*5.5 The dysfunctional couple gets in the way of shared sexuality*	P1, P4, P5, P14, P17, P18, P20

Following Newberry (Newberry, 2011; Table 5) describes the prevalence of themes and associated sub-themes in the interviews. The most significant “units of meaning” and sentences from the interviews (P1-P20) are given in the text, along with the interested person’s gender (M = Male, F=Female) and age in brackets. E.g., Patient 6 (P6-M-25y.o.). Table 6 provides a summary of the key information about interviews and patients.

**Table 6:** Information about interviews and patients.

Patient	Age	Sex	Type of cancer	Prevalence in the themes	Length of the interview
P1	32	Male	Synovial Sarcoma	5	12 min
P2	22	Male	Osteosarcoma	4	10 min
P3	22	Female	Sarcoma Ewing	6	11 min
P4	27	Male	Sarcoma Ewing	5	12 min
P5	33	Female	Giant cell tumor	6	11 min
P6	25	Male	Osteosarcoma	3	10 min
P7	21	Female	Synovial Sarcoma	6	13 min
P8	32	Male	Myxoid Liposarc	3	10 min
P9	31	Male	Undiff Pleom Sarc	4	17 min
P10	36	Male	Clear Cell Sarc	4	12 min
P11	38	Female	Sarcoma Ewing	3	13 min
P12	33	Female	Sarcoma	3	11 min
P13	35	Male	Spindle-Cell Sarc	3	10 min
P14	34	Female	Epithelioid Sarc	3	12 min
P15	35	Male	Synovial Sarcoma	5	10 min
P16	24	Male	Sarcoma Ewing	7	14 min
P17	24	Male	Osteosarcoma	8	10 min
P18	36	Female	Myxoid Liposarc	4	10 min
P19	29	Male	Osteosarcoma	3	19 min
P20	22	Male	Synovial Sarcoma	7	12 min

#### 3.2.1 Overall domain

For AYA patients with sarcoma, information, personal reflection, and relationships are key. Thematic analysis highlighted that patients need support to cope with the impact of cancer. Having good relationships and receiving information from healthcare professionals can promote sexual wellbeing.

#### 3.2.2 Theme 1

Cancer bursts into patients’ lives, forcing them to redefine their priorities. The specter of cancer brings loneliness, uncertainty about the future and anxiety, as well as the conditioning associated with the fear of “not making it,” particularly in the early stages of the disease: “there is a sword of Damocles over your head, the possibility of relapse, so you cannot even put too much, too much on your problems” (P8, M 32 y.o.). Sexuality can be neglected and pushed to one side, particularly during active treatment and when emotional relationships are insecure: “At that time, relationships and sexuality were definitely at the bottom of my list” (P1, M, 32 y.o.). Eight patients experienced stability of sexual desire during the clinical pathway: “I have not had to deal with this issue” (P10, M, 36 y.o.). The issue is not raised by seven patients whose clinicians are seen as incapable of fully processing and understanding the experience of the disease, compared to their peers: “The only person who can understand you is someone who is like you” (P3, F, 22 y.o.).

#### 3.2.3 Theme 2

Ten patients find the topic of sexuality taboo and believe that clinicians often underestimate its importance: “There is a kind of minimization of everything that is not strictly treatment” (P8-M-32y.o.). Some health professionals have an empathic relationship with their patients, encouraging them to explore this area and become aware of the implications of their “changing body”: “The nurse, being in close contact with the patient, is perhaps able to perceive the difficulties a little more than a doctor, right?. So say yes, either a psychotherapist or a nurse” (P9, M, 31 y.o.) For seven patients, health professionals facilitate an improvement in the couple’s experience of sexuality.: “It is not you who has to say: “Look, there is a risk they’ll amputate my leg,” it is not me who has to look at you” (P3, F, 22 y.o.).

#### 3.2.4 Theme 3

The scar can alter the body image of people who have faced and overcome cancer. Mutilations and physical disabilities can have a negative impact on sexuality and self-image, hindering the normal development of sexuality in six patients: “The issue of sexuality is very closely linked to the aesthetical side” (P3, F, 22 y.o). Conversely, changing the way people think about illness can help them to cope with their mutilated bodies: “Understanding how your body has changed, and how best to live with the condition for which you have been treated” (P2, M, 22 y.o.).

#### 3.2.5 Theme 4

At the time of diagnosis, five AYA patients with cancer postponed the development of their sexuality compared to their healthy peers: “There were other needs, other concerns, I did not feel the need to deal with the issue of my sexuality” (P2, M, 22 y.o.). The physical changes caused by cancer and medical treatment necessitate a re-evaluation of sexual functioning in the context of a damaged body: “During chemotherapy I had cysts, I thought I needed time to ejaculate and have a healthy channel to masturbate.” (P1, M, 32 y.o.). Five patients pointed out that during their pathway, healthcare professionals focused on fertility preservation rather than sexuality. “Sexuality is an issue that was not been addressed by the team. At first I did not think it was important to preserve sperm, I did it out of scruples and I am happy that it was suggested to me” (P9, M, 31 y.o.). At the end of treatment and during follow-up, once they have been relieved of the initial burden of cancer, AYA patients express satisfaction at having taken on the challenge of fertility preservation as a viable option for future parenthood.

#### 3.2.6 Theme 5

In the early stages of the disease, 10 patients need to discuss their condition with healthcare professionals, without the potential “interference” of their partners: “The partner is perceived as an obstacle to the freedom of expression, and there is a fear of “upsetting the partner.” (P19, M, 29 y.o.).

Sharing the illness and its impact on sexuality with a partner can lead to feelings of embarrassment and shame, not least due to the limitations of a “changed body.” After the initial personal processing, however, functional relationships can cope with the changes and challenges of illness and treatment, opening up new possibilities for partners: “There can be more dialogue, more comparison”(P16, M, 24 y.o.). Additionally, a strong relationship with a partner can enhance a patient’s coping mechanisms. However, five patients expressed a need to protect their partner, which increased mutual isolation. Conversely, in dysfunctional relationships, patients may choose to end the relationship and face their challenges alone: “Cutting the ties was, let us say, a decision to protect him, I did not want him to go through the same things I did with such a burden that it could have affected his daily life” (P20, M, 22 y.o.).

## 4 Discussion

Bone and Soft Tissue Sarcomas are a heterogeneous group of rare neoplasms, representing approximately 1% of solid tumors in all age groups (Drabbe et al., 2021). Their heterogeneity and rarity can lead to diagnostic delays, with advanced-stage diagnosis and a significant impact on QoL (Drabbe et al., 2021). Furthermore, the impact of intensive treatments, consisting of a combination of surgery, chemotherapy, and radiotherapy, can pose a considerable challenge, especially in AYA patients, who are already at a physiologically crucial stage of their life (Cherven et al., 2024,; Neylon et al., 2023). In particular, the sexual experience of Adolescents and Young Adults (AYA) patients with sarcoma is strongly influenced by biological factors (e.g., side effects of therapies, hormonal changes, neuropathy), psychological factors (e.g., anxiety, depression and body image issues) and relational factors (e.g., support from a partner and quality of communication with healthcare professionals) (Kosir et al., 2020). The diagnosis of cancer often causes patients to avoid discussing the sexual aspects of their condition, in line with theoretical models of coping with stressful situations (Lazarus and Folkman, 1984). Additionally, the side effects of cancer treatments, including fatigue, sexual and fertility problems, gastrointestinal disorders, alopecia, cardiotoxicity, skin reactions, edema, and more, as well as the invasiveness of surgery, can all reduce sexual desire and the ability to experience sexuality as pleasurable (Janssen et al., 2021).

According with recent studies (Lehmann et al., 2022; Mütsch et al., 2019) the phenomenological analysis (Husserl, 1967) of sexuality and sexual wellbeing in AYA patients with sarcoma conducted in the present study, highlighted that, although sexuality and sexual wellbeing are central to the psychological and relational wellbeing of these patients, these dimensions are often neglected in cancer care. A sarcoma diagnosis is a traumatic event that disrupts the normal psychoaffective and social development of AYAs patients and requires them to redefine their individual priorities and life plans: *“Maybe when the patient has achieved a certain stabilization and has processed the fact that he has a tumor, that he has to go through a process. you have to go through hell, but more or less you have a goal”* (P12, F, 33 y.o.). Analysis of the interviews confirmed that, in the biopsychosocial sense defined by World Health Organization (2025), sexuality is often overlooked by patients, particularly in the early stages of the disease when the focus is primarily on treatment and survival. Conversely, patients find it easier to maintain a stable sexual and emotional life when the disease has not caused major physical changes: *“For people who may not have a physical disability, who may have to sit or be bedridden for a few months, but, I do not know, maybe they have had an operation for a sarcoma in an arm or another part of the body, and in any case it does not cause that kind of physical disability, maybe they do not see a difference in that kind of situation”* (P9, M, 31 y.o.). Their altered and wounded bodies caused anxiety and distress, forcing them to approach their sexuality differently, as highlighted in recent studies (Veneroni et al., 2020; Soanes and White, 2018). However, the patient who underwent amputation showed no greater prevalence of themes and sub-themes in the interview. Twelve patients disinvested from sexuality as a result of the disease’s impact, from diagnosis through to the various stages of treatment. It is therefore important to understand how to support AYA patients and survivors in moving from disinvestment to a healthy recovery of sexual health and QoL. A recurring insight that emerged from the interviews was that many patients were concerned about the lack of open communication regarding sexuality between patients and healthcare providers: *“I think it is important to talk about it immediately, so that you believe, that is, you are aware that this problem can arise and exists. If you do not talk about it, you are not aware that it can happen, so you think it’s a subjective thing when it happens. There should be a bit more proactivity on the part of the team to bring it up because I think it is more difficult for patients to talk about it if they are not prompted to do so”* (P8, M, 32 y.o.). In line with a recent study on women with breast cancer (Zimmaro et al., 2020), two patients would have appreciated encouragement from the care team to explore these issues. The stigma still associated with this topic, together with the elusiveness of the cancer experience (Kosir et al., 2020), may lead patients to refrain from explicitly asking for information about sexuality, for fear of being judged or of asking about something that is not perceived as a priority by the care team. By contrast, in line with international literature (Albers et al., 2020; Bentsen et al., 2024; Cherven et al., 2024; Kosir et al., 2020), almost all patients (*n* = 16) thought that discussing sexuality with clinicians would have been extremely helpful, given the significant physical and psychological impact of the disease on this area. However, recent studies suggest that several barriers prevent health professionals from addressing these emotionally charged issues. These barriers include embarrassment, a perceived lack of time, a lack of training and competence in this area, concern about embarrassing patients, and the prioritization of other aspects of cancer care (Reese et al., 2017). This approach can sometimes lead to a “*conspiracy of silence*,” which increases patients’ discomfort and insecurity, and limits their development or recovery. In this context, peer support and exchange can be an effective resource for AYA patients, highlighting the need for greater involvement of patient organizations in this area, as suggested by Douvrel et al. (2025): “*Only we patients can understand each other. It’s bad to say, but it’s true, no matter how hard the doctors try, they will never understand what it means to feel like you are losing your hair, they will never understand what it means not to have a leg*” (P3, F, 22 y.o.). The lack of communication about these issues may be perceived by patients as clinicians minimizing their condition, as shown by the results of this study: *“There is a kind of minimization of everything that is not strictly treatment. This lack of information about everything that is ancillary, has led… quite rightly, to a focus on the disease without considering everything else around it”* (P8, M, 32 y.o.). In line with recent studies (Kosir et al., 2020), respondents identified psychologists and nurses as the professionals with whom they felt more comfortable opening up, due to their skills and availability: *“I believe that the staff must also be qualified in this area in order to be able to give the right advice. I believe that the staff must also be qualified in this area in order to be able to give the right advice. Certainly a nurse, but I think the best professional to talk to about these things is a psychologist”* (P20, M, 22 y.o.); *“The nurse, being in close contact with the patient, can maybe perceive the difficulties a little bit more than a doctor, right? The doctor, I do not know, I see him here a little bit more detached from the patient, a little bit for ehh, I always thought also a little bit for self-defense, a little bit for… they see so many”* (P9, M, 31 y.o.). In this context, a multidisciplinary approach could promote sexual education and awareness of sexual health issues among cancer patients.

The integration of a new body image emerges as a central issue in the experience of AYA patients with sarcoma, particularly for those who have undergone amputation or invasive surgery. Adapting to a mutilated body is a dynamic process that can have a profound effect on self-esteem and the quality of close relationships. In line with recent studies on this topic (Gozzi et al., 2022), for many patients the scar symbolizes a dual process: for some, they signify resilience and success in overcoming cancer; for others, they hinder the development of a new body image and sexual identity. In this sense, scar integration can help to reconstruct the cancer experience and develop a new self-image. These are areas in which psychological support and open dialogue with health professionals can be challenging (Soanes and White, 2018).

Patients have different opinions on the right time to talk about sexuality, which highlights how personal subjectivity can influence the choice of the best time to do so: “*I think it’s a very subjective thing and that there’s no right or wrong time to bring it up when you are in trouble*” (P1, M, 32 y.o.). Consistent with a recent study (Albers et al., 2020), addressing sexuality at the outset of cancer treatment may help some patients cope with the uncertainty of a cancer diagnosis and an “*uncertain future*.” However, several patients felt that the “right” time to discuss sexual wellbeing was at the end of treatment, when they were more relaxed and able to return to “*normal*” life: “*Probably at the end of treatment, on your return to normal life*” (P14, F, 34 y.o.) Nevertheless, many patients felt that sexuality should be consistently addressed throughout the care pathway to increase engagement and proactivity, in line with extensive international literature (Lehmann et al., 2022; Mütsch et al., 2019).

The impact of sarcoma on sexuality inevitably affects the quality of emotional relationships and the perception of the partner’s role in the care process at all ages. However, this impact is particularly significant in AYAs compared with older adults (Drabbe et al., 2021). Marital dynamics play a crucial role in promoting or hindering sexual recovery. Relationships based on open communication and the ability to adapt to change are more beneficial than relationships characterized by avoidance and unspoken behaviors. In line with recent literature (Liberacka-Dwojak et al., 2024), involving partners in discussions with the healthcare team could enhance intimacy and couple cohesion for certain patients, thereby preventing feelings of shame and fear of judgment from their partners, which some patients experience when treatments make them feel *“tired, weaker and psychologically think they cannot cope”* (P16, M, 24 y.o). Discussing these sensitive topics with the support of the healthcare team could help patients overcome their burden and sexual dysfunction. It could also help them renegotiate their sexual preferences and self-image, which would have a positive impact on their QoL (Kosir et al., 2020): *“I am not the one who has to give you the news, I am not the one who has to look at you when I give you the news, I am not the one who has to prepare my speech so that you understand that it is not an obstacle for me and I hope it is not an obstacle for you”* (P3, F, 22 y.o.). Some patients choose to face cancer alone in order to protect their partner from the emotional burden of the disease, and to prevent unnecessary strain on their relationship. However, the analysis highlighted the importance of the patient initially processing the new condition alone, before involving their partner at a later stage. Few AYA patients see their partner as a real barrier to discussing sexuality with the clinician, due to the embarrassment this might cause, and because sexuality is experienced as a deeply personal matter. Without a solid relationship, it can be difficult to start a new one after active treatment ends due to discomfort and “*performance anxiety*”: *“I do not have the emotional and psychological strength to start a new relationship and therefore to deal with sexuality”* (P14, F, 34 y.o.). Furthermore, similar recent studies (Lehmann et al., 2025), have shown that for some AYA patients, the topic of sexuality is confined to an interest in fertility. This is also addressed by clinicians, albeit sometimes in a fleeting and fragmentary way “*to safeguard the future development project*” (Sub-themes 4.3).

Finally, the results of the descriptive analysis of the functional and symptomatic scales suggest that some people may have difficulty managing their emotions and adapting to their daily roles. This highlights the need for psychological, occupational and social support. Overall, quality of life (QoL) showed associations with almost all dimensions of the scale, confirming that the overall wellbeing of these patients depends on several factors simultaneously (World Health Organization, 2025; Drabbe et al., 2021). The descriptive analysis of distress suggests a significant impact of the disease on QoL.

### 4.1 Recommendations for further research

The results of this preliminary, in-depth exploration could provide a valuable starting point for further research on this complex topic. The research group aims to carry out this research through a longitudinal study in order to capture changes in the field of sexuality over time. It is actually an ongoing qualitative pilot study exploring the opinions and perspectives of healthcare professionals on communicating about sexuality with AYA patients with sarcoma. The present qualitative study should be considered a “pilot study” that will necessarily need to be expanded in the future through larger multicenter studies, in order to validate the identified themes with a larger sample and a more balanced gender distribution. The authors believe that this perspective could provide valuable insights to inform effective support interventions for this population.

### 4.2 Implications for policy and practice

Improving knowledge of the sexual health and psycosexual needs of AYA patients with sarcoma is essential for implementing personalized interventions that can enhance clinical pathways and patient survivorship. Healthcare professionals should recognize the importance of sexuality and sexual wellbeing to the QoL of AYA patients with cancer, and implement strategies to promote and enhance dialogue about these issues.

### 4.3 Limitations

Despite the interesting findings of the thematic analysis, the authors acknowledge the limitations of the study. The study, which was conducted among Italian AYA patients, could have been influenced by cultural factors, beliefs, and specific attitudes toward sexuality. This could affect the generalizability of the results. However, generalizability is not the primary objective of qualitative and phenomenological approaches (Sandelowski, 2000; Husserl, 1967), which aim to explore a phenomenon in depth to fully understand patients’ experiences which may differ across cultures. In this sense, the authors continued to collect and analyze data until they reached data saturation (Tuckett, 2004; Sandelowski, 1995). Furthermore, Lincoln and Guba’s criteria were employed to enhance the study’s rigor and reliability (Lincoln and Guba, 1985). The variability of content related to sexuality over time could have been better addressed through a longitudinal study. However, the authors believe that a qualitative design is the most appropriate approach for exploring patients’ experiences and needs in relation to sexuality. Although the authors aimed to maximize variability in the sample, they recruited an imbalanced sample in terms of gender: 13 men and seven women. The greater participation of men may reflect their greater willingness to discuss issues related to sexuality, as suggested by previous research (Collaço et al., 2025). In this regard, although gender differences were not the primary focus of the present study, sociocultural norms may have influenced participants’ willingness to participate in the research, reflecting their differing concerns about openly discussing sexual desire, sexual needs, or sexual wellbeing. However, to counteract this potential bias, the study’s interview team was gender-balanced (two men and two women), in order to facilitate the participation of both women and men in discussions on a particularly sensitive topic such as sexuality. Similarly, excluding AYA patients under the age of 18 from the study may have prevented the authors from capturing important aspects of the phenomenon. Given the complexity of obtaining informed consent from minors’ parents or legal guardians, the study included participants between the ages of 18 and 39 at the time of data collection. Therefore, the results are based on a retrospective assessment of the experiences of adolescent or young adult patients at the time of diagnosis and active treatment, who were between the ages of 18 and 39 at the time of data collection. Furthermore, the authors considered the development of sexuality and related care needs in this age group to be worthy of further study. Finally, the EORTC QLQ-C30 questionnaire (Aaronson et al., 1993) was used for the aims of this study. Although it is a validated tool, it has not been specifically validated for AYAs. A separate module for AYAs with cancer was subsequently developed by Sodergren et al. (2025) and explores domains not covered by the EORTC QLQ-C30. However, final validation of this instrument is currently underway, and the inclusion of descriptive quantitative instruments (EORTC QLQ-C30 and the Distress Thermometer) has been used to provide basic clinical and psychological context regarding the sample’s overall levels of distress and QoL.

## 5 Conclusion

In line with recent studies (Janssen et al., 2021; Kosir et al., 2020), the phenomenological analysis conducted to explore sexuality in AYA patients with sarcoma highlighted the severe impact of cancer on the self-perception, emotional relationships and quality of sexual wellbeing of these young patients. In line with our findings, recent studies suggest that a lack of information and communication with healthcare professionals, coupled with the taboo surrounding sexuality in oncology, are significant barriers to the psychophysical wellbeing of patients with cancer (Albers et al., 2022). Improving psychological support and information through sexual education interventions within a multidisciplinary team, involving partners where appropriate, could promote a more positive and conscious management of sexuality in cancer care (Carter et al., 2018). Recent studies (De Leo et al., 2024) emphasize that the relationship with nurses is a key element in improving the engagement and satisfaction of cancer patients. Finally, an integrated approach that considers sexuality as an essential aspect of QoL and overall wellbeing for AYA patients with sarcoma could enhance care pathways and QoL for this sensitive population.

## Data Availability

The raw data supporting the conclusions of this article will be made available by the authors, without undue reservation.
